# Tensile Behaviors and Strain Hardening Mechanisms in a High-Mn Steel with Heterogeneous Microstructure

**DOI:** 10.3390/ma15103542

**Published:** 2022-05-15

**Authors:** Shengde Zhang, Yanke Liu, Jian Wang, Shuang Qin, Xiaolei Wu, Fuping Yuan

**Affiliations:** 1 State Key Laboratory of Nonlinear Mechanics, Institute of Mechanics, CAS, 15 Beisihuan West Road, Beijing 100190, China; zhangshengde@imech.ac.cn (S.Z.); liuyanke@imech.ac.cn (Y.L.); wangjian2@imech.ac.cn (J.W.); xlwu@imech.ac.cn (X.W.); 2 School of Engineering Science, University of Chinese Academy of Sciences, 19A Yuquan Road, Beijing 100049, China

**Keywords:** microband-induced plasticity, heterogeneous structures, steels, strain hardening, strengthening, ductility

## Abstract

Heterogeneous structures with both heterogeneous grain structure and dual phases have been designed and obtained in a high-Mn microband-induced plasticity (MBIP) steel. The heterogeneous structures show better synergy of strength and ductility as compared to the homogeneous structures. Higher contribution of hetero-deformation induced hardening to the overall strain hardening was observed and higher density of geometrically necessary dislocations were found to be induced at various domain boundaries in the heterogeneous structures, resulting in higher extra strain hardening for the observed better tensile properties as compared to the homogeneous structures. MBIP effect is found to be still effective in the coarse austenite grains of heterogeneous structures, while the typical Taylor lattice structure and the formation of microband are not observed in the ultra-fine austenite grains of heterogeneous structures, indicating that decreasing grain size might inhibit the occurrence of microbands. High density of dislocation is also observed in the interiors of BCC grains, indicating that both phases are deformable and can accommodate plastic deformation. It is interesting to note that the deformation mechanisms are highly dependent on the phase and grain size for the present MBIP steel with heterogeneous structures.

## 1. Introduction

Steels with high strength and excellent ductility/toughness are always desirable in steel industries [1,2,3,4,5,6,7]. As a result, several types of advanced steels have been developed in the past several decades for automotive industries, such as dual-phase steels [8,9], transformation-induced plasticity (TRIP) steels [10,11,12], and twinning-induced plasticity steels (TWIP) [13,14,15]. The strength and ductility of steels are highly dependent on the chemical composition, the constituent phases, and the deformation mechanisms, while a high combination of strength and ductility (i.e., tensile strength × elongation) is always required for advanced automotive applications.

A significant research interest has been dedicated to the Mn austenitic steels particularly during the last two decades due to their high tensile strengths with exceptional ductility [10,11,12,13,14,15,16,17,18,19,20,21,22,23,24,25]. The deformation mechanisms for the Mn austenitic steels are highly dependent on the Mn content. Martensite transformation is the dominant strain hardening mechanism when the Mn content is low (about 5–12%) in the Mn austenitic steels due to the resultant ultra-low stacking fault energy (SFE) [10,11,12]. While the deformation mechanism is gradually transited from TRIP effect to TWIP effect with increasing Mn content in the Mn austenitic steels [13,14,15]. TRIP and TWIP effects [10,11,12,13,14,15] have been two of the most promising deformation modes due to their high work hardening capacity. Deformation twins (DTs) start to form in favorably oriented grains after the initial plastic deformation with a few percent by slip in the TWIP steels, and such dynamic microstructure refinement by DTs can result in the reduction of the dislocation mean free path in the matrix for strain hardening, the so-called “dynamic Hall-Petch (DHP) effect” [13,14,15]. Moreover, several physical-based models have been proposed to describe the contributions from both the isotropic and kinematic hardening mechanisms and understand the property-microstructure relationship in TWIP steels [23,24,25,26,27,28].

In the past decade, a new high-Mn steel has been developed with a new strain hardening mechanism, namely, microband-induced plasticity (MBIP) effect [16,17,18,19,20,21,22]. This mechanism has been achieved in Fe-Mn-Al-C alloys with high SFE (50–90 mJ/m^2^). Several investigations have revealed that the Al addition of about 10 wt.% into the high Mn (about 28–30 wt.%) austenitic steels is beneficial for not only remarkable weight reduction but also superior tensile properties (ultimate tensile strength of ~900 MPa and total elongation of ~100%) comparable to or even better than those of TWIP steels [16,17,18,19,20,21,22]. The strain hardening by MBIP effect has been attributed to the formation of the in-grain narrow shear zones bounded by geometrically necessary dislocations (GNDs) [20,22,29,30]. Moreover, other dislocation substructures, such as highly dense dislocation walls (HDDWs) and Taylor lattices, have also been reported to contribute to the strain hardening [20,22,30].

A limitation of metals and alloys with coarse grains (CGs) for restricting their industrial applications is their low yield strength. Cold working and grain refinement have been extensively utilized to obtain high density of dislocation and ultrafine grains (UFGs) for strengthening metals and alloys by severe plastic deformation (SPD) methods [31,32,33]. While the elevation in strength by this way generally results in a significant reduction in ductility [34]. Thus, SPD methods followed by partial recrystallization have also been applied to obtain heterogeneous grain structures (composed of both CGs and UFGs), resulting in increased yield strength and appreciable ductility in various types of metals and alloys [35,36,37,38,39,40,41,42,43,44]. The high ductility in heterogeneous grain structures was attributed to the hetero-deformation induced (HDI) hardening associated with the plastic incompatibility between the soft domains and the hard domains [45,46]. Thus, it might be possible to achieve a better synergy of strength and ductility in MBIP steels with heterogeneous microstructures. Until now, the MBIP effect was only identified in these Fe-Mn-Al-C alloys with CGs, whether or not the MBIP effect is effective in the UFGs or the heterogeneous grain structures is still an open question. Moreover, the HDI hardening effects were not discovered in these MBIP steels with heterogeneous grain structures, in which HDI hardening would be a much more complex process. The HDI hardening in MBIP steels with heterogeneous grain structures would come from the interaction between CGs and UFGs in the initial microstructure, as well as the interaction between the CG matrix and the possibly formed microbands inside CGs during tensile deformation.

In this regard, a series of uniaxial tensile tests and load-unload-reload (LUR) tests, along with detailed microstructure characterizations prior to and after tensile testing, have been conducted in the present study to investigate the deformation behaviors in a MBIP steel (1.00 C, 28 Mn, 10 Al, 0.001 P, 0.0037 S and the balance of Fe in wt.%) with various microstructures, so as to illustrate the grain-size dependence of MBIP effect, as well as the effect of heterogeneous grain structures (HDI hardening) on tensile properties.

## 2. Materials and Methods

The MBIP steel used in the present study has a chemical composition of 1.00 C, 28 Mn, 10 Al, 0.001 P, 0.0037 S, and the balance of Fe (all in wt.%). The materials first were melted in an induction furnace under protection of Ar atmosphere. Then the ingots with a diameter of 30 mm were hot-extruded into rods with a diameter of 22 mm at 1173 K. The rods have a full austenitic phase after being annealed at 1473 K for 2 h and immediately quenched in water (such sample was named as CG_1_). In the SPD procedure, the rod first was subjected to the equal-channel angular pressing (ECAP) for 1 pass, thus a large strain was introduced into the ECAP sample and the size of rod was unchanged. In order to further increase the deformation degree of sample, the sample was subsequently cold-rolled to a final thickness reduction of 41.6% and hereinafter was specified as ECAP + CR. In addition, such sample with a final thickness of 7 mm was designed in consideration of the fracture tests in our future work.

**Figure 1:**
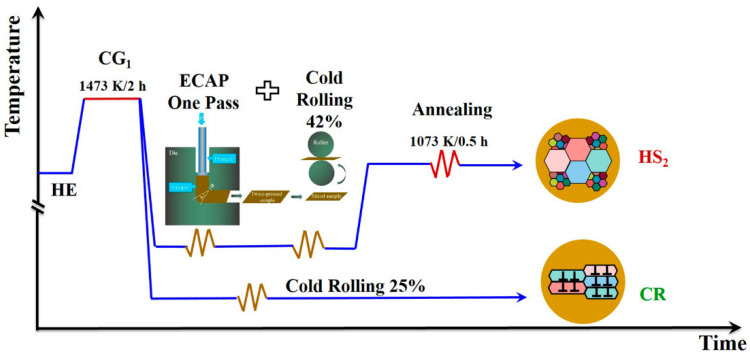
Schematic diagram of procedures for processing materials.

In order to obtain various microstructures, the rods were processed by SPD methods and then followed by the heat treatments. Specifically, samples were annealed at 1053, 1073, 1273, and 1373 K temperatures for 30 min (these samples were named as HS_1_, HS_2_, CG_3_ and CG_2_, respectively), and at a temperature of 1073 K for 15 min and 45 min (these samples were named as HS_3_ and HS_4_, respectively). As a comparison, some samples with homogeneous structures were obtained only by cold-rolled with a thickness reduction of 25% on the CG_1_ samples (such sample was specified as CR).The procedures for processing materials are shown in Figure 1.

Dog bone plate samples, which have 10 mm gage length, 2.5 mm gage width, and 1.2 mm gage thickness, were used for the tests. All tensile and LUR tests were conducted using an Instron 5966 testing machine at a strain rate of 5 × 10^−4^/s under room temperature. Strain was measured by an extensometer during tensile deformation. Displacement-control mode was applied, so as to ensure the same strain rate during unloading-reloading period. Details for LUR tests can be referred to our previous papers [41,43]. Electron backscattered diffraction (EBSD), transmission electron microscope (TEM), and high-resolution electron microscope (HREM) were utilized to reveal the microstructures before and after tensile testing. The details for sample preparation of EBSD and TEM can be found elsewhere [44].

## 3. Results and Discussions

**Figure 2:**
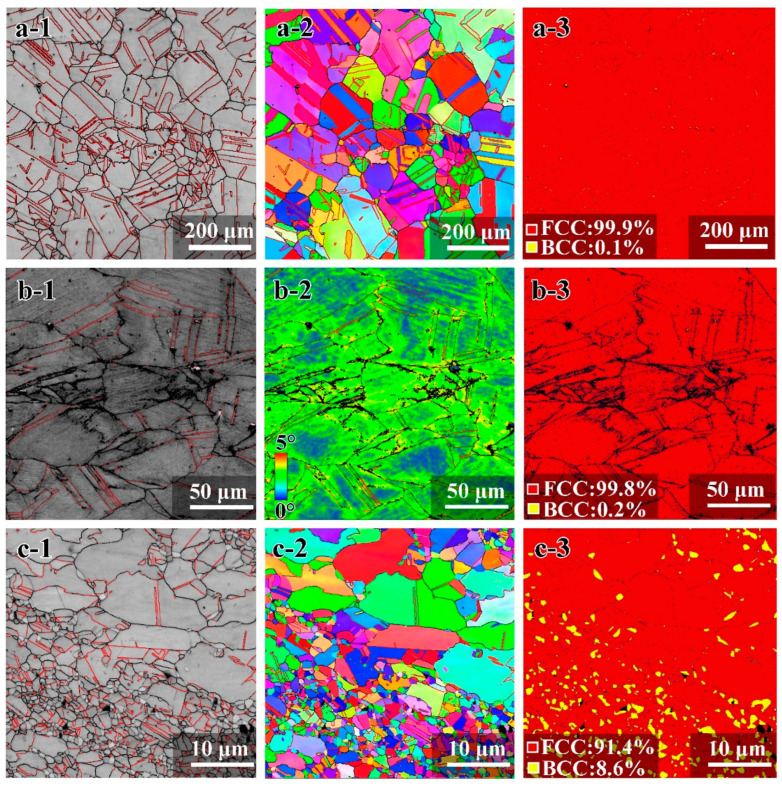
The EBSD images for the CG_1_ sample: (**a-1**) Image with high-angle grain boundaries (GBs) and TBs; (**a-2**) Inverse Pole Figure (IPF) image; (**a-3**) Phase image. The EBSD images for the CR sample: (**b-1**) Image with high-angle GBs and TBs; (**b-2**) KAM image; (**b-3**) Phase image. The EBSD images for the HS_2_ sample: (**c-1**) Image with high-angle GBs and TBs; (**c-2**) IPF image; (**c-3**) Phase image.

**Figure 3:**
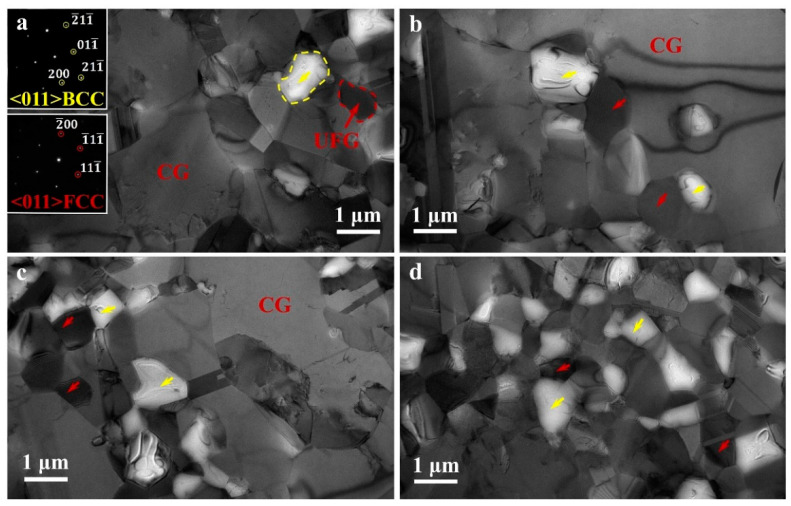
TEM observations for the HS_2_ sample prior to tensile testing. (**a**) Both FCC and BCC phases can be observed; (**b**,**c**) the interiors of both FCC and BCC grains are relatively clean, indicating a low density of dislocation; (**d**) the BCC grains are distributed non-uniformly. Red arrows: the UFGs of FCC phase; yellow arrows: the UFGs of BCC phase.

The microstructure characterizations by EBSD for the annealed sample at 1473 K for 2 h, the CR sample, and the sample after SPD and being annealed at 1073 K for 30 min are shown in Figure 2. The annealed sample at 1473 K has equi-axed CGs with an average grain size of 183 μm (Figure 2(a-1,a-2)); numerous annealing twins and twin boundaries (TBs) can also be observed in the annealed CG structure, thus this sample is named as CG_1_ sample. The CG_1_ sample has a single phase of FCC austenite (Figure 2(a-3)). The CR sample has the typical elongated lamella structure along the rolling direction (Figure 2(b-1)), while the CR sample displays a high density of dislocation in the grain interiors as indicated by the high kernel average misorientation (KAM) value in Figure 2(b-2). The CR sample also displays a single austenitic phase (Figure 2(b-3)). A heterogeneous grain structure is found for the HS_2_ sample (Figure 2(c-1,c-2)), in which both CGs (tens of μm) and UFGs (about 1 μm or less than 1 μm) are observed. Moreover, this sample displays a dual-phase structure, in which the austenite phase has a volume fraction of about 91.4%, and the volume fraction of the BCC phase is about 8.6%. Thus, this sample is a typical heterogeneous structure, and numerous annealing twins and TBs are also found in the grain interiors of austenite grains for the HS_2_ sample.

TEM observations for characterizing the detailed microstructures for the HS_2_ sample have been conducted, and these images are displayed in Figure 3. Both FCC and BCC phases can be observed, and these two phases can be identified by the selected area diffractions (SADs) in the insets of Figure 3a. The average size of BCC grains is about 1 μm or less than 1 μm. While, both CGs (~10 μm) and UFGs (about 1 μm or less than 1 μm) can be observed for the austenite phase, indicating a heterogeneous grain structure for the austenite phase. The interiors of both FCC and BCC grains are relatively clean, indicating a low dislocation density. The BCC grains are found to be distributed non-uniformly, some areas are observed to have a high-volume fraction of BCC phase (Figure 3d).

**Figure 4:**
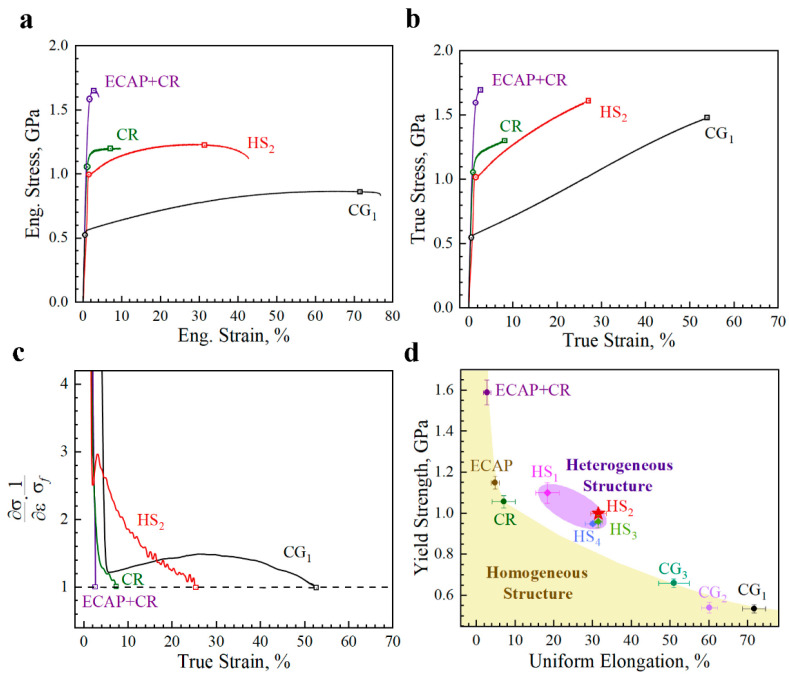
The tensile properties. (**a**) Tensile engineering stress–strain curves for typical microstructures. (**b**) Tensile true stress–strain curves for typical microstructures. (**c**) The curves of normalized hardening rate as a function of true strain for typical microstructures. (**d**) The YS as a function of UE for all tested samples.

First, uniaxial quasi-static tensile tests were conducted on samples with various microstructures at a strain rate of 5 × 10^−4^ /s under room temperature. The engineering stress–strain curves and the corresponding true stress–strain curves for the typical three samples (the CG_1_, CR, and HS_2_ samples) are shown in Figure 4a,b, respectively. In Figure 4a,b, the yield strength (YS) points are marked by circles, the ultimate tensile strength (UTS) and uniform elongation (UE) points are marked by squares. The CG_1_ structure has a very low YS of about 523 MPa while a high UE of about 71.5%. The CR sample has a YS of about 1054 MPa, while the UE is very low (about 7.2%). The YS is about 992 MPa and the UE is about 31.5% for the HS_2_ sample. The HS_2_ sample has a similar YS (992 MP vs. 1054 MPa) while has a much higher UE (31.5% vs. 7.2%), as compared to the CR sample. The normalized hardening rate ($\left(\partial \sigma /\partial \varepsilon \right)/\sigma_{f}$) is plotted as a function of true strain for these three samples in Figure 4c. It is shown that the normalized hardening rate of the HS_2_ sample is much higher than that of the CR sample, and even higher than that for the CG_1_ sample at some strain range. The hardening rate curve for the HS_2_ sample displays a transient up-turn phenomenon, which is the typical behavior for heterogeneous structures [40,41]. The curves for YS as a function of UE for all tested samples are displayed in Figure 4d. It is observed that the HS_2_ samples show a better synergy of strength and ductility over the homogeneous samples.

**Figure 5:**
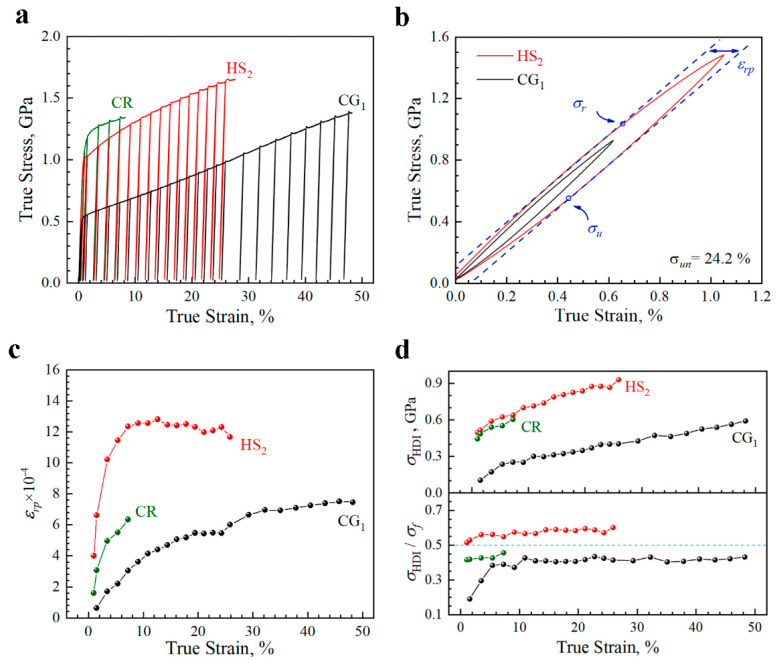
HDI hardening for the typical samples. (**a**) True stress–strain curves for LUR tests. (**b**) Typical hysteresis loops. (**c**) $\varepsilon_{\mathrm{rp}}$ as a function of true strain. (**d**) $\sigma_{\mathrm{HDI}}/\sigma_{f}$ and $\sigma_{\mathrm{HDI}}$ as a function of true strain.

It has been indicated that the superior tensile properties in the heterogeneous structures can be attributed to the HDI hardening [40,45,46], which can be induced by the plastic deformation incompatibility between hard and soft domains. In this regard, in order to illustrate and compare the HDI hardening effect on the tensile properties for various samples (the CG_1_, CR, and HS_2_ samples), the corresponding LUR tests have been conducted and the true stress–strain curves for LUR tests are displayed in Figure 5a. The typical hysteresis loops are shown in Figure 5b. Moreover, the width of hysteresis loop is generally characterized by the residual plastic strain ($\varepsilon_{\mathrm{rp}}$) on each unloading and a larger $\varepsilon_{\mathrm{rp}}$ represents a stronger back stress (HDI stress, $\sigma_{\mathrm{HDI}}$) [43]. The loop widths of typical samples are plotted as a function of true strain in Figure 5c. It is shown that $\varepsilon_{\mathrm{rp}}$ for the HS_2_ sample is much higher than those for the CG_1_ and CR samples, indicating a higher contribution of $\sigma_{\mathrm{HDI}}$. In addition, the $\sigma_{\mathrm{HDI}}$ can be calculated by the average value of the unloading yield stress and the reloading yield stress ($\sigma_{\mathrm{HDI}}=\left(\sigma_{u}+\sigma_{r}\right)/2$) from the hysteresis loops of LUR tests, based on the method proposed in our previous research [45]. Then, $\sigma_{\mathrm{HDI}}$ and the fraction of HDI stress on the overall flow stress ($\sigma_{\mathrm{HDI}}/\sigma_{f}$) are plotted as a function of true strain for these samples in Figure 5d. Generally, $\sigma_{\mathrm{HDI}}/\sigma_{f}\;$can be considered as a contribution of HDI hardening to the overall strain hardening. It is clearly shown that $\sigma_{\mathrm{HDI}}/\sigma_{f}\;$for the HS_2_ sample is much higher than those for the CG_1_ and CR samples. This indicates that the HDI hardening plays a more important role in the heterogeneous structures than in the homogeneous structures, which could be the origin of the better tensile properties in the heterogeneous structures.

**Figure 6:**
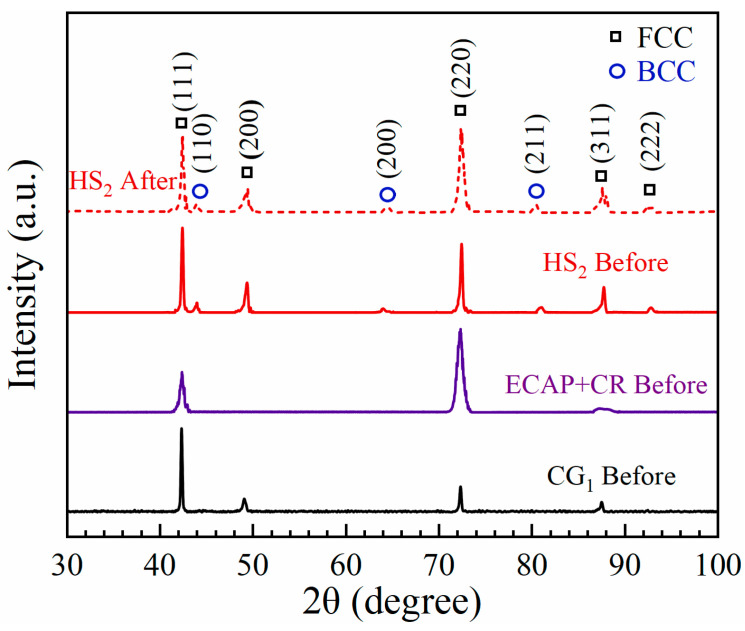
The XRD spectra for various samples prior to and after tensile testing.

In order to identify the phases and the corresponding volume fractions for various phases prior to and after tensile testing, the XRD spectra for the aforementioned three samples (the CG_1_, CR, and HS_2_ samples) are displayed in Figure 6. It is clearly indicated that both CG_1_ and CR samples have a single FCC phase, while a dual-phase structure (FCC and BCC phases) is observed in the HS_2_ sample prior to tensile testing, and these observations are consistent with previous EBSD results. The integrated peak areas for different phases can be used to estimate the volume fraction of each phase. Hence, the relative volume fractions of various phases (FCC and BCC phases) for the HS_2_ sample prior to and after tensile testing are estimated. It is observed that the volume fractions of both phases are nearly unchanged, indicating no phase transformation during tensile deformation for the HS_2_ sample.

**Figure 7:**
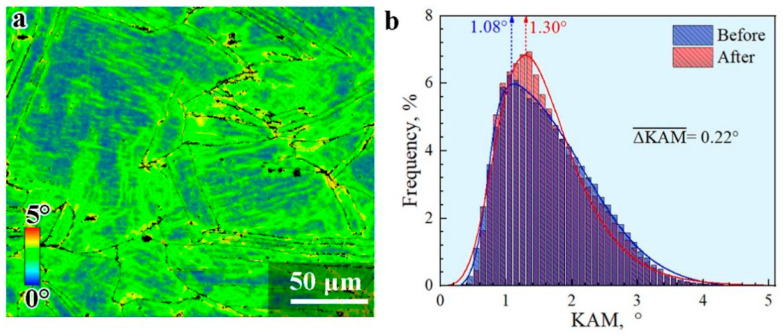
The microstructure characterization by EBSD for the CR sample after tensile testing. (**a**) KAM mapping. (**b**) Histogram distributions of KAM value.

The HDI hardening is generally accommodated by the GNDs at the boundaries of hard/soft domains. The density of GNDs can generally be reflected by the KAM value based on the strain gradient theory and the method proposed by Gao and Kubin [47,48]:$\;\rho_{\mathrm{GND}}=2\theta /lb$. Where, θ represents the misorientation at local points, *l* is the unit length (400 nm in the present study) for the local points, and *b* is the Burger’s vector for the materials (4.56 nm for the current steel). Thus, the KAM mapping after tensile deformation for the CR sample is displayed in Figure 7a. Moreover, the histogram distributions of KAM value prior to and after tensile testing for the CR sample are shown in Figure 7b. Then, the average KAM values prior to and after tensile testing for the CR sample are estimated to be about 1.08° and 1.30°, respectively. The increment of GND density after tensile deformation for the CR sample is estimated to be about 2.41 × 10^14^/m^2^.

**Figure 8:**
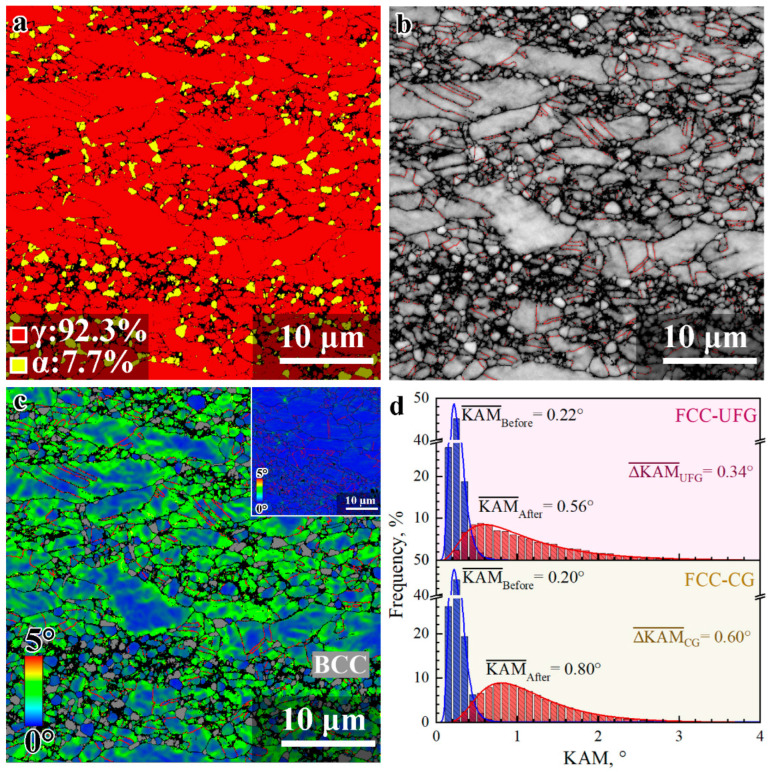
The microstructure characterization by EBSD for the HS_2_ sample after tensile testing. (**a**) Phase map. (**b**) Image with high-angle GBs and TBs. (**c**) KAM mapping, Inset: KAM mapping prior to deformation. (**d**) Histogram distributions of KAM value.

The phase map by EBSD for the HS_2_ sample after tensile deformation is also shown in Figure 8a. The volume fractions of both phases (FCC and BCC phases) are similar to those prior to tensile testing, which is consistent with the XRD results. It is also shown that some TBs are still observed after tensile deformation (Figure 8b). The observation in Figure 8b indicates that the grains are refined and the average grain size is reduced after tensile deformation, resulting in strong strain hardening for the HS_2_ sample, similar to the DHP effect for TWIP steels [13,14,15]. In the HS_2_ sample, GNDs can be produced at the phase boundaries, as well as at the boundaries of CGs and UFGs. The KAM mapping after tensile deformation for the HS_2_ sample is displayed in Figure 8c. Moreover, the histogram distributions of KAM value prior to and after tensile testing for the HS_2_ sample are shown in Figure 8d. Then, the average KAM values prior to and after tensile testing for the CGs in the HS_2_ sample are estimated to be about 0.20° and 0.80°, the increment of KAM value (0.60°) is much higher than that for the UFGs (0.34°). The increment of GND density after tensile deformation for the CGs in the HS_2_ sample is estimated to be about 6.56 × 10^14^/m^2^, which is much higher than that (3.72 × 10^14^/m^2^) for the UFGs in the HS_2_ sample. Moreover, the increments of GND density of both CGs and UFGs in the HS_2_ sample are much higher than that of the CR sample. These results indicate that the heterogeneous structure can produce a larger strain gradient and higher density of GNDs during tensile deformation, resulting in higher HDI hardening for better tensile properties.

**Figure 9:**
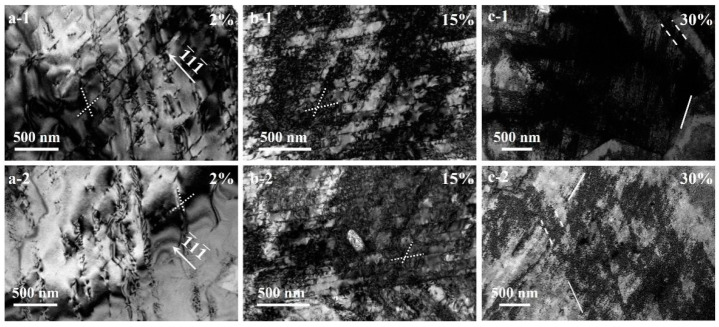
TEM observations for the HS_2_ sample showing deformation mechanisms in the coarse grains of austenite phase at varying interrupted tensile strain: (**a-1**,**a-2**) 2%; (**b-1**,**b-2**) 15%; (**c-1**,**c-2**) 30%. Dotted lines: slip traces; solid lines: single-wall DBs; dash lines: microbands.

**Figure 10:**
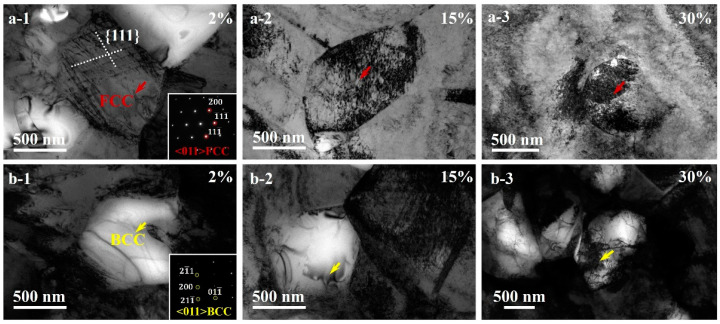
TEM observations for the HS_2_ sample showing deformation mechanisms in the UFGs of austenite phase: (**a-1**) 2%; (**a-2**) 15%; (**a-3**) 30%. TEM observations for the HS_2_ sample showing deformation mechanisms in the grains of BCC phase at varying interrupted tensile strain: (**b-1**) 2%; (**b-2**) 15%; (**b-3**) 30%.

TEM observations for characterizing the microstructure evolution during the tensile deformation for the HS_2_ sample have been conducted, and these images are displayed in Figure 9 and Figure 10. The dominant deformation mechanism is highly dependent on the Mn content and the SFE in the Mn austenitic steels, transiting from TRIP effect to TWIP effect, and finally to MBIP effect with increasing Mn content and SFE [16,17,20,22,30]. The MBIP effect has been proved to be effective in high Mn (about 28–30 wt.%) austenitic steels with homogeneous CGs [16,17,20,22,30]. Here, we check whether or not this MBIP effect is still effective in the present high Mn steels (high SFE of 50–90 mJ/m^2^) with heterogeneous grain structures and dual phases. TEM images showing deformation mechanisms in the CGs of austenitic phase at varying interrupted tensile strains are shown in Figure 9a. At a small tensile strain of 2% (Figure 9(a-1,a-2)), the deformation substructures in the CGs of austenite phase are characterized by dislocations pile up and slip along principle planar gliding planes, resulting in continuous increase of strain hardening rate. Additionally, the dislocations glide on {111} planes are beneficial to form the Taylor lattices (TLs), which is a relative low energy structure as compared to the dislocation cells generated by the dislocation wavy slips [30]. Thus, some typical TLs are found in the CGs of austenite phase. At a tensile strain of 15% (Figure 9(b-1,b-2)), no obvious cell wall is observed, but TLs become more intensive and the interval of slip traces becomes much finer. Meanwhile, some TLs rotate moderately in order to activate other slip systems for further accommodation of plastic deformation, as indicated by the obscure slip traces in Figure 9(b-1,b-2). At a large tensile strain of 30% (Figure 9(c-1,c-2)), single-wall domain boundaries (DBs) with high density of GNDs are formed due to the rotation of misoriented TLs domains. Subsequently, the dislocation walls paralleling to the existed single-wall DBs are formed. Such paired-wall dislocation structures constitute the microband. Thus, the deformation substructures in the CGs of austenite phase are characterized by both the formation of single-wall DBs with high density of GNDs and microbands with paired well-defined boundaries. The formation of DBs, microbands, and their intersections with each other provide strong strain hardening. These observations indicate that MBIP effect is still effective in the CGs of heterogeneous grain structure, and the strain hardening for the CGs can still be attributed to the glide plane softening effect, the formation of microbands and substructures with high density of GNDs [16,17,20,22,30].

TEM images showing deformation mechanisms in the UFGs of austenite phase and the grains of BCC phase at varying interrupted tensile strains are displayed in Figure 10. In the UFGs of austenite phase, planar dislocations slip along the principle gliding planes to form the TLs which is also observed at a small tensile strain of 2% (Figure 10(a-1)). However, the formation of DBs or microbands is not observed at larger tensile strains, although high density of dislocation is found in the grain interiors of UFGs (Figure 10(a-2,a-3)). These observations indicate that the MBIP effect is not effective in the UFGs, and the decreasing grain size might inhibit the occurrence of microbands. At a small tensile strain of 2% (Figure 10(b-1)), a few dislocations are observed in the grains of BCC phase. Moreover, the number of dislocations increases with increasing tensile strains, and high density of dislocation is observed in the interiors of BCC grain (Figure 10(b-2,b-3)). These observations indicate that both phases are deformable and can accommodate plastic deformation, while the deformation mechanisms are varied based on the phases and the grain sizes.

## 4. Conclusions

In the present study, heterogeneous structures have been obtained in a high-Mn steel by cold rolling and subsequent critical-temperature annealing processes. Then, the tensile properties of the heterogeneous structures have been investigated and compared with the homogeneous structures by only cold rolling process; the corresponding deformation mechanisms have also been revealed. The main findings are summarized as follows:

(1) The HS sample shows a dual-phase structure, and has both coarse grains and ultrafine grains. The HS sample has a similar yield strength with the CR sample, while the uniform elongation of the HS sample is much larger than that of the CR sample. Overall, the heterogeneous structures display a better synergy of strength and ductility over the homogeneous structures.
(2) The HS sample has a higher contribution of hetero-deformation induced hardening to the overall strain hardening, as compared to the CR sample. Hetero-deformation induced hardening was found to play a more important role in the heterogeneous structures than in the homogeneous structures. The heterogeneous structures were found to produce a higher strain gradient and higher density of geometrically necessary dislocation during tensile deformation. The produced high density of geometrically necessary dislocation at various domain boundaries and the extra hetero-deformation induced hardening should be the origins of the observed better tensile properties in the heterogeneous structures.
(3) The typical Taylor lattice structure, the formation of single-wall domain boundaries and microbands are still observed in the austenitic CGs of heterogeneous structures, although they are found to be no longer effective in the austenitic UFGs of heterogeneous structures. Thus, MBIP effect is found to be grain-size dependent, and the occurrence of microbands might be prohibited with decreasing grain size. High density of dislocation is observed in the interiors of both FCC and BCC grains, and both phases can accommodate plastic deformation. It is interesting to note that the deformation mechanisms are highly dependent on the phase and grain size for the present MBIP steel with heterogeneous structures. The present findings should provide insights into the deformation mechanisms and the engineering applications for the high-Mn steels with heterogeneous structures.

## Data Availability

Not applicable.
